# Allele mining, amplicon sequencing and computational prediction of *S*olanum *melongena* L. *FT*/*TFL1* gene homologs uncovers putative variants associated to seed dormancy and germination

**DOI:** 10.1371/journal.pone.0285119

**Published:** 2023-05-03

**Authors:** Ranjita Subramaniam, Vijay Subbiah Kumar

**Affiliations:** Biotechnology Research Institute, Universiti Malaysia Sabah, Jalan UMS, Kota Kinabalu, Sabah, Malaysia; USDA-ARS Southern Regional Research Center, UNITED STATES

## Abstract

The *FT*/*TFL1* gene homolog family plays a crucial role in the regulation of floral induction, seed dormancy and germination in angiosperms. Despite its importance, the *FT*/*TFL1* gene homologs in eggplant (*Solanum melongena* L.) have not been characterized to date. In this study, we performed a genome-wide identification of *FT*/*TFL1* genes in eggplant using *in silico* genome mining. The presence of these genes was validated in four economically important eggplant cultivars (Surya, EP-47 Annamalai, Pant Samrat and Arka Nidhi) through Pacbio RSII amplicon sequencing. Our results revealed the presence of 12 *FT*/*TFL1* gene homologs in eggplant, with evidence of diversification among *FT*-like genes suggesting their possible adaptations towards various environmental stimuli. The amplicon sequencing also revealed the presence of two alleles for certain genes (*SmCEN*-1, *SmCEN*-2, *SmMFT*-1 and *SmMFT*-2) of which *SmMFT*-2 was associated with seed dormancy and germination. This association was further supported by the observation that seed dormancy is rarely reported in domesticated eggplant cultivars, but is commonly observed in wild species. A survey of the genetic regions in domesticated cultivars and a related wild species, *S*. *incanum*, showed that the alternative allele of *S*. *incanum* was present in some members of the Pant Samrat cultivar, but was absent in most other cultivars. This difference could contribute to the differences in seed traits between wild and domesticated eggplants.

## Introduction

*Flowering Locus T* (*FT*)/*Terminal Flower1* (*TFL1*) gene homologs are important regulators of flowering time, a fundamental process in angiosperms which involves a morphologically complex shift from vegetative to reproductive development in plants [1]. This process has a direct impact on crop yield [2] and has been the focus of much research, particularly on genetic modifications of flowering time and flowering responses for improved crop productivity [3]. Gene duplications in the *FT/TFL1* genes have resulted in multiple paralogs with diversified functions rarely reported. Plants utilize them as an intrinsic strategy to refine floral responses towards various environmental and endogenous signals [4]. Acquiring an in-depth understanding of the *FT*/*TFL1* gene family is crucial in expediting the development of new cultivars that possess desirable characteristics, such as improved flowering time and enhanced productivity [4, 5]. The ideal timing of flowering is crucial especially in the midst of seasonal progressions to ensure that seeds are set under suitable conditions and to maximize their chances of survival [6]. Multiple endogenous and exogenous signals are integrated into various pathways to regulate flowering time. For example, in the model plant *Arabidopsis thaliana*, the floral initiation is modulated via a number of pathways which include photoperiod, ambient temperature, age, vernalization, autonomous and hormonal pathways [7]. These routes converge in the key integrators like mobile florigen *Flowering Locus T* (*FT*) [8].

*FT* is a member of the Phosphatidylethanolamine Binding Protein (PEBP) gene superfamily which is highly conserved across bacteria, yeast, plants to mammals with diversified functions. For an instance, PEBP proteins in animals participate in the controlling of cell growth and differentiation. In plants, they act as key players in the floral transition along with other developmental processes. PEBP gene families are basically grouped into three main clades in angiosperms: *Flowering Locus T* (*FT*), *Terminal Flower 1* (*TFL1*) and *Mother of FT and TFL1* (*MFT*). Based on the discovery of *MFT*-like genes in both basal and land plants, it is postulated that they are the evolutionary ancestor to both *FT*-like and *TFL1*-like genes as these gene clusters are only observed in gymnosperms and angiosperms [9]. In *Arabidopsi*s, six members representing these three clades have been identified where two genes, *FT* and *Twin Sister of FT* (*TSF*) being *FT*-like genes, *TFL1*, *Arabidopsis Centroradialis Homologue* (*ATC*) and *Brother of FT and TFL1* (*BFT*) being *TFL*-like genes, while, *MFT* being placed under the *MFT*-like clade [10]. Although these genes share high sequence similarities, they diverged enough to play antagonistic roles as either floral promoters or repressors. *FT* and *TFL1* proteins are small and mobile and are involved in transcriptional regulation but do not possess DNA binding domain [11]. *FT* interacts with bZIP transcription factor *FD* through 14-3-3 proteins and thus, promotes floral initiation by activating floral meristem identity genes as in *Apetala 1* (*Ap1*) and *Suppressor of Overexpression Of Constans 1* (*SOC1*) in the shoot apical meristem (SAM) [12].

On the other hand, the *FT* paralog, *TSF*, promotes flowering redundantly with *FT* but shows distinct floral activation under short-day conditions [13]. *MFT* also acts redundantly with *FT* in flowering time regulation where overexpression of the gene results in slightly early flowering while loss-of-function mutation was aphenotypic [14]. The characterization of *MFT* homologs in several plants exhibited different roles in flowering time regulation. For an instance, *MFT* homologs reported to have no effect in the flowering transition in species such as *Populus nigra* [15], *Glycine max* [16], *Citrus latifolia* [17] and *Picea abies* [18]. Furthermore, *MFT* in *Dendrobium nobile* [19] and *Hevea brasiliensis* [20] delayed flowering time. *MFT* homologs have critical role playing in seed dormancy and germination. In *Arabidopsis*, *MFT* negatively regulates germination under far-red light conditions while strongly promotes seed dormancy [21]. Similarly, in *Triticum aestivum MFT* functions as negative regulator of seed germination and positive regulator of dormancy [22]. Converse to *FT*-like genes, in *Arabidopsis*, *TFL1* maintains the indeterminate plant architecture and also induces delay in flowering transition. *ATC*, the *TFL1* paralog shows functional redundancy with *TFL1* and acts as a floral inhibitor in short-day conditions [23]. Furthermore, *BFT* is suggested to mimic *TFL*-like activity and functions redundantly with *TFL1* in regard to inflorescence meristem development and inhibits floral transition under high salinity environment [24].

Besides manoeuvring flowering processes, *FT*/*TFL1* gene families participate in various indispensable crop developmental events. Recent reports show that *FT*-like proteins have been involved in tuberization in potato [25], cessation of meristem growth in tomato [26], stomatal control in *Arabidopsis* [27], bulb formation in onion [28], plant architecture in maize [29], among others. The *FT* paralogs in various species reflected diversified responses of each paralog within respective species to different environmental and endogenous cues. For an example, in rice, *Heading Date 3A* (*HD3A*), an *FT* ortholog triggers flowering under short-day (SD) conditions. Meanwhile, *Rice Flowering Locus T 1* (*RFT1*) exerts function as a floral promoter under LD as well SD conditions [10]. In order to expand explorations of *FT*/*TFL1* genes, genetic variations in these gene families have been proven to accelerate innovations in the traits governed by these genes. In tomato, combinations of allelic variations in *FT* and *TFL1* genes have been exploited to optimize flowering signals and thus, to increase the crop productivity [2]. Similar approach can be conducted on eggplant. However, eggplant *FT*/*TFL1* gene homologs are not known and have not been characterized.

Cultivated eggplant ranks as the third most important crop species in Solanaceae, following tomato and potato [30]. Eggplant has a global production of around 58.6 million tons in 2021 [31]. Eggplant supplements various nutrients into human diet such as fibers, proteins, vitamins, minerals, phenylpropanoid compounds, antioxidants and so on [32]. Eggplant is particularly a photoperiod-insensitive plant [33]. Generation of advanced germplasms improved yield is one of the major breeding objectives in eggplant [34]. With this goal in mind, we have performed an extensive *in silico* mining of the genes from multiple eggplant genomes and have also extended the search for allelic variations in four commercially important cultivars using PacBio’s long reads amplicon sequencing approach. Here, we have characterised the *FT*/*TFL1* gene homologs in eggplant and provide new insights into their functions and potential applications in eggplant breeding.

## Materials and methods

### *In silico* mining of *FT/TFL1* gene homologs from eggplant genome assemblies

To identify homologs of the *FT/TFL1* gene, we conducted a BLAST survey using *FT/TFL1* coding sequences from various plant species as queries against three publicly available eggplant genome assemblies. The collections of coding sequences from the ’Nakate-Shinkuro’ cultivar [35], eggplant line ’67/3’ [36], and cultivar HQ-1315 [37] were used and referred to as Sme_r2.5.1, *S*. *melongena*-67/3, and *S*. *melongena*-HQ, respectively. A consensus sequence with 100% identity was generated by comparing the gene sets extracted from the three genomes. The resultant nucleotide sequences were converted into protein sequences using the Fgenesh gene prediction tool (http://www.softberry.com/) and further annotated using a BLASTp analysis. The gene structure predictions from Sme_r2.5.1 and *S*. *melongena*-HQ were utilized, and the coding sequences were compared with the corresponding genomic sequences from the parental scaffolds of the genome assemblies to validate exon/intron boundaries. This process was also complemented by manual curation.

### Sequence alignment and phylogenetic analysis

The amino acid sequences of *FT*/*TFL1* gene sequences from various plant species were downloaded from NCBI non-redundant database (https://www.ncbi.nlm.nih.gov). Multiple sequence alignment was carried out with ClustalW using default parameters. A neighbour-joining phylogenetic tree was constructed with Molecular Evolutionary Genetics Analysis software version 10.2.6 [38], using the Poisson model with gamma-distributed rates. The nodal reliability in the phylogenetic tree was evaluated by 10,000 bootstrap replicates.

### Functional domain and promoter analysis

To investigate the evolutionary relationships among *FT* homologs from different plant species, an alignment of the amino acid sequences was performed with a focus on conserved regions at exon II (position 85) and segment B of exon IV (positions 128–141). We included sequences from eggplant, *Arabidopsis*, onion, sugar beet, longan, soybean, sunflower, tobacco, sugarcane, Norway spruce and tomato. Variations in the critical motifs of eggplant *FT* homologous regions were analysed via sequence comparisons. In addition, the upstream regions (~ 8 kb) of the start codon (referring to translational initiation site) which could potentially cover the promoter regions of the *FT* paralogs were analysed for the presence of any transposon fragments via NCBI BLASTn survey. The protein sequences for the *FT/TFL1* gene family were sourced from The Arabidopsis Information Resource (TAIR), GenBank and Phytozome as described previously [1]. The sequences were manually verified to ensure accuracy.

### Plant materials

Eggplant cultivars namely, Surya, EP-47 Annamalai, Pant Samrat and Arka Nidhi were procured from the World Vegetable Center (AVRDC). The corresponding AVRDC accessions for the cultivars were VI045276, VI047336, VI045550 and VI045274, respectively. The freshly obtained seeds were cultivated in the glasshouse at Biotechnology Research Institute, Universiti Malaysia Sabah to compare variations among *FT*/*TFL1* homologs among the cultivars. Three biological replicates representing each cultivar were used for the downstream applications.

### Amplicon generation, SMRT library preparation, and PacBio sequencing

The genomic DNA of eggplant cultivars was extracted from leaf samples using a modified CTAB-based method [39]. Six regions, *SmTFL*1, *SmCEN*-1, *SmCEN*-2, *SmCEN*-4, *SmMFT*-1, and *SmMFT*-2, were amplified in three biological replicates for each cultivar. An asymmetric barcode system (i.e. different combination of barcodes attached to both forward and reverse primers) was utilized to assign unique barcode combinations to the homologs in each plant. The barcodes were introduced to the amplicons through a two-step PCR protocol according to the Barcoded Universal Primer workflow (https://www.pacb.com). PCR1 consists of primers which were tagged with universal and gene-specific sequences and used with genomic DNA template. The second step, PCR2, introduces barcodes to the amplicons by performing PCR on the amplicon template generated in PCR1. A list of the primers used is available in S1–S3 Tables. Note that this multiplexed amplicon sequencing also includes homologs extracted from mutant populations of the aforementioned cultivars.

The two-step PCR was conducted using Kapa HiFi HotStart ReadyMix PCR kit. Reactions consisted of 1x reaction buffer (Kapa HiFi HotStart ReadyMix), 0.3 μM of forward primers, 0.3 μM of reverse primer, approximately 50–100 ng of genomic DNA in a 25 μl total volume. Cycle parameters were ~1 min/1 kb gene at 95°C, followed by 30 cycles of 20 seconds at 98°C and 15 seconds at 60°C, and a 1 min/1 kb gene at 72°C followed by a final extension of 1 min/1 kb gene at 72°C. Barcodes were attached to the amplicons in the second round of PCR with identical conditions except that 10–20 ng of DNA template (amplicons from the 1st round of PCR) was used.

All the amplicons were pooled in equimolar amounts and the pooled sample was purified with 1.0x volume of AMPure PB Beads (Beckman-Coulter Woerden, the Netherlands) before eluting in 37 μl of elution buffer. SMRTbell library was constructed from the pooled amplicons with a starting amount of 1.26 μg of the sample pool, following the standard procedures for SMRTbell adapter ligation. Sequencing of the libraries was conducted with standard procedures using P6v2C4 chemistry (Pacific Biosciences, California, USA) with six hours of movie time.

### Pacbio sequence data processsing

The sequencing data files in the format of.h5 files were converted into.bam files by using the bax2bam program (version 0.0.9). The demultiplexing of the.bam files was performed using the pblima program (version 1.11.0). Finally, the phased amplicon sequences were obtained through the long amplicon analysis protocol conducted in pblaa program (version 2.4.2). The program generated subread coverage for each allele, and the amplicon coverages were manually calculated as the total number of amplicons within each sample that had been sequenced. The corresponding subread identities for each allelic sequence were retrieved from one of the output files of pblaa, and the number of unique ZMWs in the pool of subreads was counted to generate amplicon coverages. The bioinformatic analysis programs were installed through Miniconda 3 (https://conda.io/miniconda.html).

Each set of gene homologs belonging to the four different cultivars were manually transferred to MEGA v10.2.6 [38] and aligned using ClustalW program to screen for nucleotide variations in the alleles. The detected variants were further characterized using the Sorting Intolerant from Tolerant (SIFT) program with the UniProt-SwissProt + TrEMBL 2010_09 database under default parameters. The *MFT*-like genes were compared with the transcripts of W-4 (*S*. *incanum* L.) and the Ramnagar Giant cultivar [40]. The comparison was made using the BLASTn program with the *MFT*-like genes mined from their respective RNASeq data. The RNASeq data used for this comparison were downloaded from the National Centre for Biotechnology Information (NCBI; https://www.ncbi.nlm.nih.gov) with primary accession numbers GAYR00000000 and GAYS00000000.

## Results

### Identification of *FT/TFL1* gene homologs in *S*. *melongena*

The analysis of *FT*/*TFL1* gene homologs on three different publicly available genome sequences, SME_r2.5.1, *S*. *melongena*-67/3 and *S*. *melongena*-HQ, resulted in the discovery of several *FT*-like, *TFL1*-like and M*FT*-like gene sequences. An equal number of *TFL1*-like and *MFT*-like genes were obtained from the predicted coding sequences (CDSs) of the genome assemblies, totalling two *MFT*-like and five *TFL1*-like genes. However, the number of *FT*-like genes varied among the three eggplant genomes, with two, four and five gene sequences found in SME_r2.5.1, *S*. *melongena*-67/3 and *S*. *melongena*-HQ, respectively, as summarised in Table 1.

**Table 1:** Distribution of *FT*/*TFL1* gene homologs across three different genome assemblies.

*FT*/*TFL1* gene	SME_r2.5.1	*S*. *melongena*-67/3	*S*. *melongena*-HQ
*FT*-like genes	2	4	5
*TFL*1-like genes	5	5	5
*MFT*-like genes	2	2	2

The comparison of the coding sequences of each gene obtained from the three genome assemblies revealed that a minimum of two sequences with 100% similarity were present for each gene, providing higher confidence level to the sequences (Table 2). *SmFT*-5 was excluded from subsequent analyses as only partial CDS fragments were obtained from the mining process. The application of the gene prediction tool FGENESH on the corresponding genomic sequence produced similar results, indicating that further investigation is necessary to properly identify the gene structure of its homolog.

**Table 2:** Comparison of coding sequences of *FT*/*TFL1* gene homologs between three different genome assemblies.

Genes	SME_r2.5.1	*S*. *melongena*-67/3	*S*. *melongena*-HQ	Consensus CDS (bp)	Deduced protein (aa)
*SmFT*_1	100%	100%	100%	534	177
*SmFT*_2	100%	100%	Variant1	528	175
*SmFT*_3	-	100%	100%	534	177
*SmFT*-4	-	100%	100%	408	135
*SmMFT*-1	100%	100%	100%	522	173
*SmMFT*-2	100%	100%	100%	513	170
*SmCEN*-1	100%	100%	100%	528	175
*SmCEN*-2	100%	100%	100%	519	172
*SmCEN*-3	Partial	100%	100%	540	179
*SmCEN*-4	100%	Variant2	100%	534	175
*SmTFL1*	100%	100%	100%	528	175

**Fig 1:**
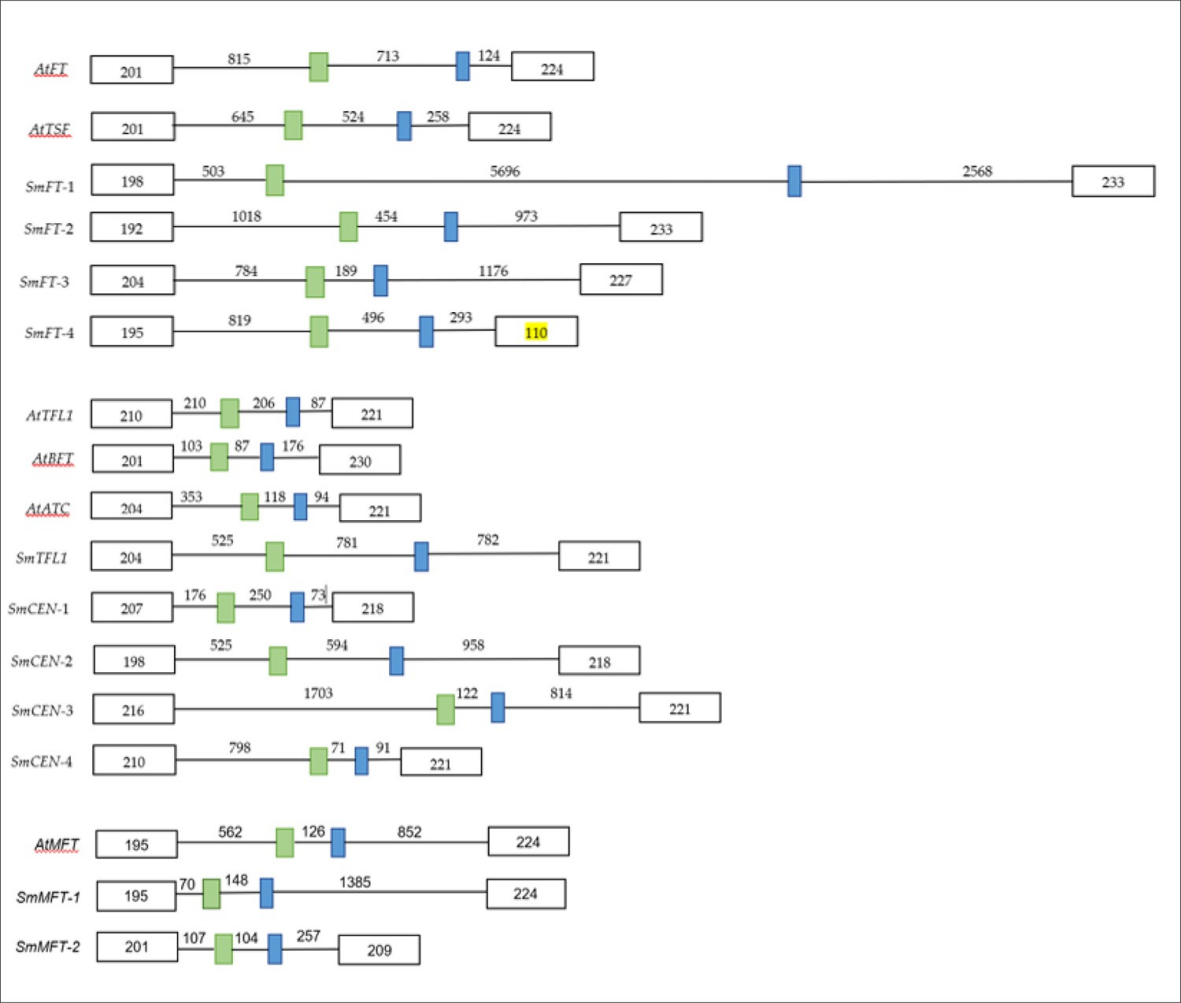
Genomic organizations of *FT/TFL1* gene homologs of *S*. *melongena* and *Arabidopsis*. The open reading frames (ORFs) are basically related to sequences derived from Sme_r2.5.1. Boxes indicate exons and lines indicate introns. Numbers represent the length (base pairs) of corresponding exons and introns. Green boxes denote exon II (62 bp) and blue boxes denote exon III (41 bp).

The gene structure of all the *FT*/*TFL1* gene homologs (except for *SmFT*-5) were consistent with the typical structures reported for this gene family i.e. four exons and three introns placed at conserved positions as seen in *Arabidopsis* [23]. However, the lengths of the introns were variable, as depicted in Fig 1. Exons I and IV had variations in lengths from 192 to 216 bp and from 209 to 233 bp, respectively. However, exon IV of *SmFT*-4 was an exception, with an unusual length of 110 bp. In contrast, exon II and exon III remained conserved in length at 62 bp and 41 bp, respectively, across all analysed genes [23].

**Fig 2:**
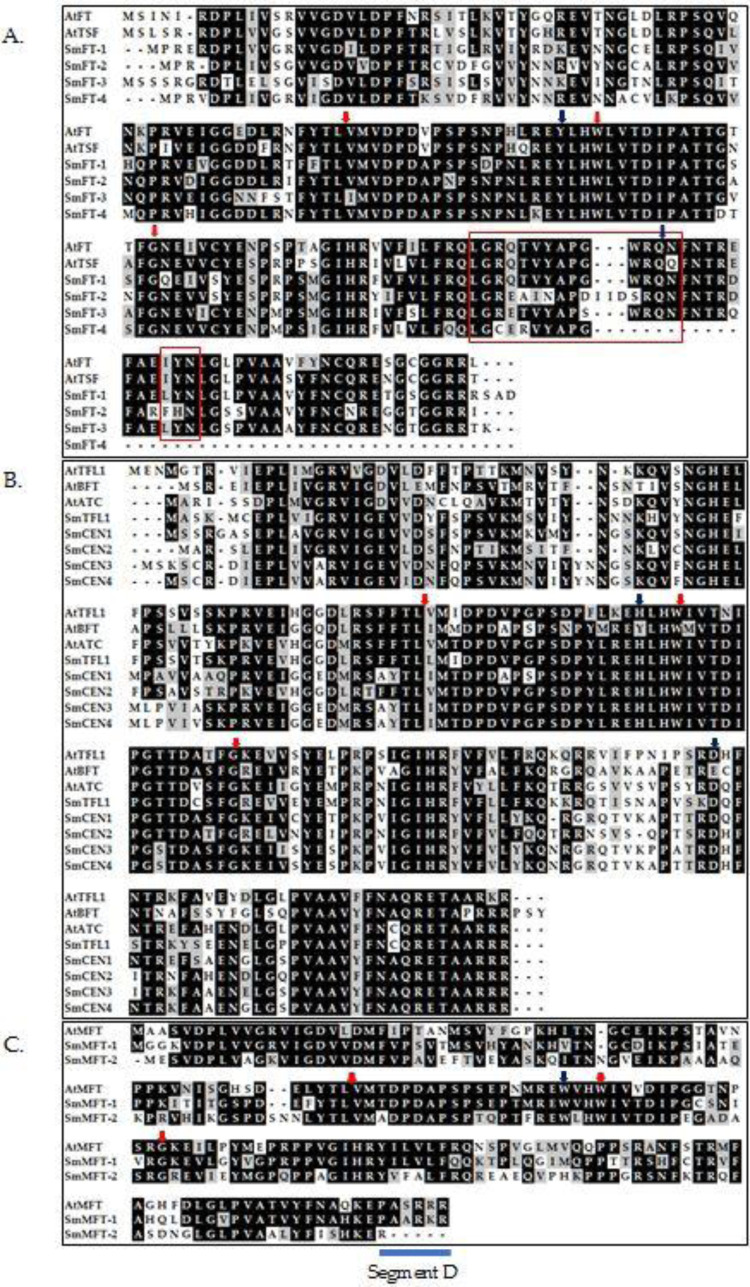
Alignments of deduced amino acid sequences of the *FT/TFL1* gene homologs of *S*. *melongena* and *Arabidopsis*. (A) Amino acid sequence alignments of *FT*-like genes; (B) Amino acid sequence alignments of *TFL*-like genes; (C) Amino acid sequence alignments of *MFT*-like genes. Gaps are represented by dots. Red arrows above sequences point at intron positions. Arrowheads in blue indicate amino acids critical to distinguish *FT*-like, *TFL*-like, or *MFT*-like proteins. Boxes in red indicate the highly-conserved amino acid sequences located in exon IV of *FT*-like proteins. The thick line indicates segment D of exon IV [41].

Further analysis of *SmFT*-4 (which had an unusual shortened length), revealed the presence of a premature stop codon, due to a single base pair mutation. From the amino acid sequence alignment (Fig 2), the codon following the stop codon was expected to be tryptophan (W), encoded by the TGG codon, but it was changed to a stop codon (TGA) due to a substitution of G to A. The remaining nucleotide sequences from the point of the stop codon encode a putative full-length coding sequence. The coding sequences of this homolog mined from the both *S*. *melongena*-HQ and *S*. *melongena*-67/3 were found to be identical.

In contrast, *SmMFT*-2 had only one residue before a stop codon in segment D, as shown in Fig 2, which was fewer, compared to other *MFT*-like genes. To investigate the expression of this gene, the transcriptome of *S*. *melongena* (cultivar Ramnagar Giant) was analyzed. A highly similar (99.8%) *MFT*-2 allele was found to be expressed in the cultivar (S1 Fig), and the downstream sequences were identical to one of the transcripts of the *MFT*-2 gene.

### Phylogenetic analysis of *S*. *melongena FT/TFL1* gene homologs

**Fig 3:**
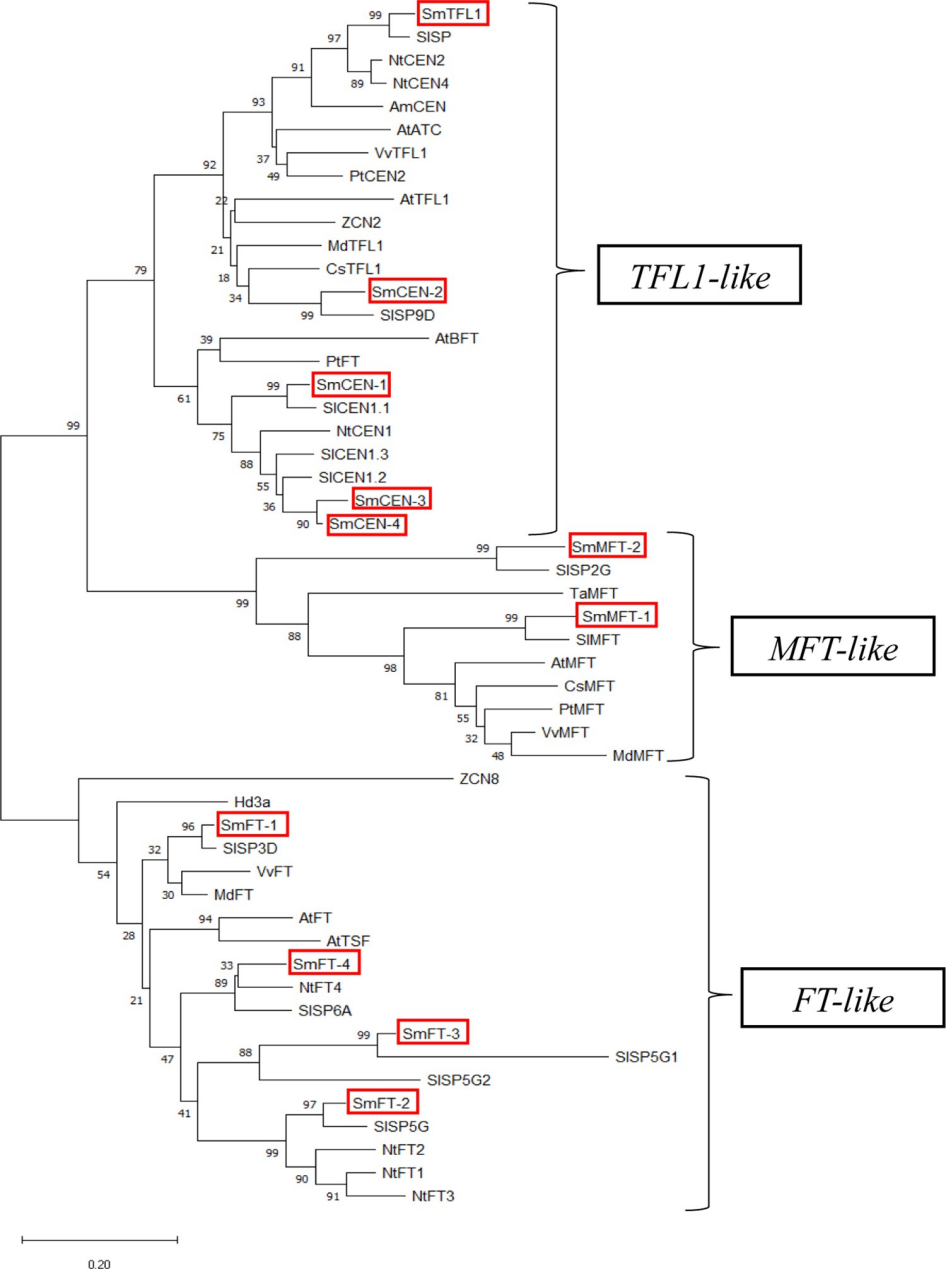
Phylogenetic tree of the *FT*/*TFL1* gene homologs in *S*. *melongena* and various angiosperms. The tree was generated using a neighbour-joining (NJ) method. The three subfamilies are shown on the right. Protein sequences with following accessions were obtained from Genbank and Solanaceae Genomics Network (solgenomics.net): *Arabidopsis thaliana FT* (BAA77838.1), *TSF* (Q9S7R5.1), *TFL1*(P93003.1), *ATC* (BAA75933.1), *BFT* (Q9FIT4.1), *MFT* (AEE29676.1); *Vitis vinifera FT* (ABL98120.1), *TFL1* (AAM46142.), *MFT* (XP_003634198.1); *Triticum aestivum MFT* (BAK78908.1*); Citrus sinensis MFT* (XP_006490744.1), *TFL1* (AAR04683.1); *Populus trichocarpa FT* (EEF06030.2), *CEN2* (XP_002312811.1), *MFT* (ABC26020.1); *Malus domestica FT* (ACL98164.1), *TFL1* (NP_001280887.1), *MFT* (XP_008374830.1); *Antirrhinum majus CEN* (CAC21563.1); *Nicotiana tabacum FT1* (AFS17369.1), *FT2* (AFS17370.1), *FT3* (AFS17371.1), *FT4* (AFS17372.1), *FT5* (QCW12730.1), *CEN1* (Q9XH44.1), *CEN2* (Q9XH43.1), *CEN4* (Q9XH42.1); *Beta vulgaris FT1* (ADM92608.1), *FT2* (ADM92610.1); *Zea mays ZCN2* (ABW96225.1), *ZCN8* (ABW96231.1); *Oryza sativa Hd3a* (BAB61027.1); *Oryza rufipogon* (BAO03058.1); *Solanum lycopersicum SP2G* (Solyc02g079290), *MFT* (Solyc03g119100), *CEN1*.*2* (Solyc01g009580), *CEN1*.*3* (Solyc01g009560), *CEN1*.*1* (Solyc03g026050), *SP9D* (Solyc09g009560), *SP* (Solyc06g074350), *SP6A* (Solyc05g055660), *SP3D* (Solyc03g063100), *SP5G2* (Solyc11g008640), SP5G1(Solyc11g008660), *SP5G* (Solyc05g053850).

A neighbour-joining phylogenetic tree was constructed to analyse the phylogenetic relationships between the homologous *FT*/*TFL1* gene of *S*. *melongena* (except for *SmFT*-5) and other angiosperms. The analysis revealed the clustering of three major subfamilies: *SmFT*-1, *SmFT*-2, *SmFT*-3 and *SmFT*-4 belong to the *FT*-like subfamily; *SmTFL1*, *SmCEN*-1, *SmCEN*-2, *SmCEN*-3 and *SmCEN*-4 belong to the *TFL1*-like subfamily; and *SmMFT*-1 and *SmMFT*-2 belong to the *MFT*-like subfamily (Fig 3). The results showed that *S*. *melongena FT*/*TFL1* proteins have closer relationships with those from the same *Solanaceae* family such as *Solanum lycopersicum* and *Nicotiana tabacum*.

The putative orthologs of *FT*, *SmFT*-1, *SmFT*-2 and *SmFT*-3 showed all the characteristic features of *FT*-like protein genes. These include the conserved amino acids Tyr85 and Gln140 (Tyr84 and Gln 139 in *SmFT*-1, Tyr82 and Gln 140 in *SmFT*-2, Tyr86 and Gln 141 in *SmFT*-3, and Tyr83 and a missing Gln in *SmFT*-4). Furthermore, the highly conserved amino acid sequences in exon IV critical for *FT* activity, LGRQTVYAPGWRQN, as well as the highly conserved LYN triad, were identical in *SmFT*-1 [23]. However, minor variations in the LGRQTVYAPGWRQN were observed in *SmFT*-2 and *SmFT*-3. With regards to LYN triad, *SmFT*-1 and *SmFT*-3 exhibit complete similarities, while *SmFT*-2 displays an FHN instead. In the next subfamily, the putative orthologs of *TFL1*-like genes, *SmTFL1*, *SmCEN*-1, *SmCEN*-2, *SmCEN*-3 and *SmCEN*-4 displayed conservation in amino acid residues His88 and Asp144 in the corresponding positions (His86 and Asp 142 in *SmTFL1*, His87 and Asp142 in *SmCEN*-1, His84 and Asp139 in *SmCEN*-2, His90 and Asp146 in *SmCEN*-3, and His88 and Asp144 in *SmCEN*-4) (Fig 2). *SmMFT*-1 and *SmMFT*-2 which were grouped together in the third subfamily carried the critical amino acid residue Trp, which is distinct from Tyr and His in *FT* or *TFL1*.

### Functional domain and putative promoter analysis of *FT*-like gene homologs

**Fig 4:**
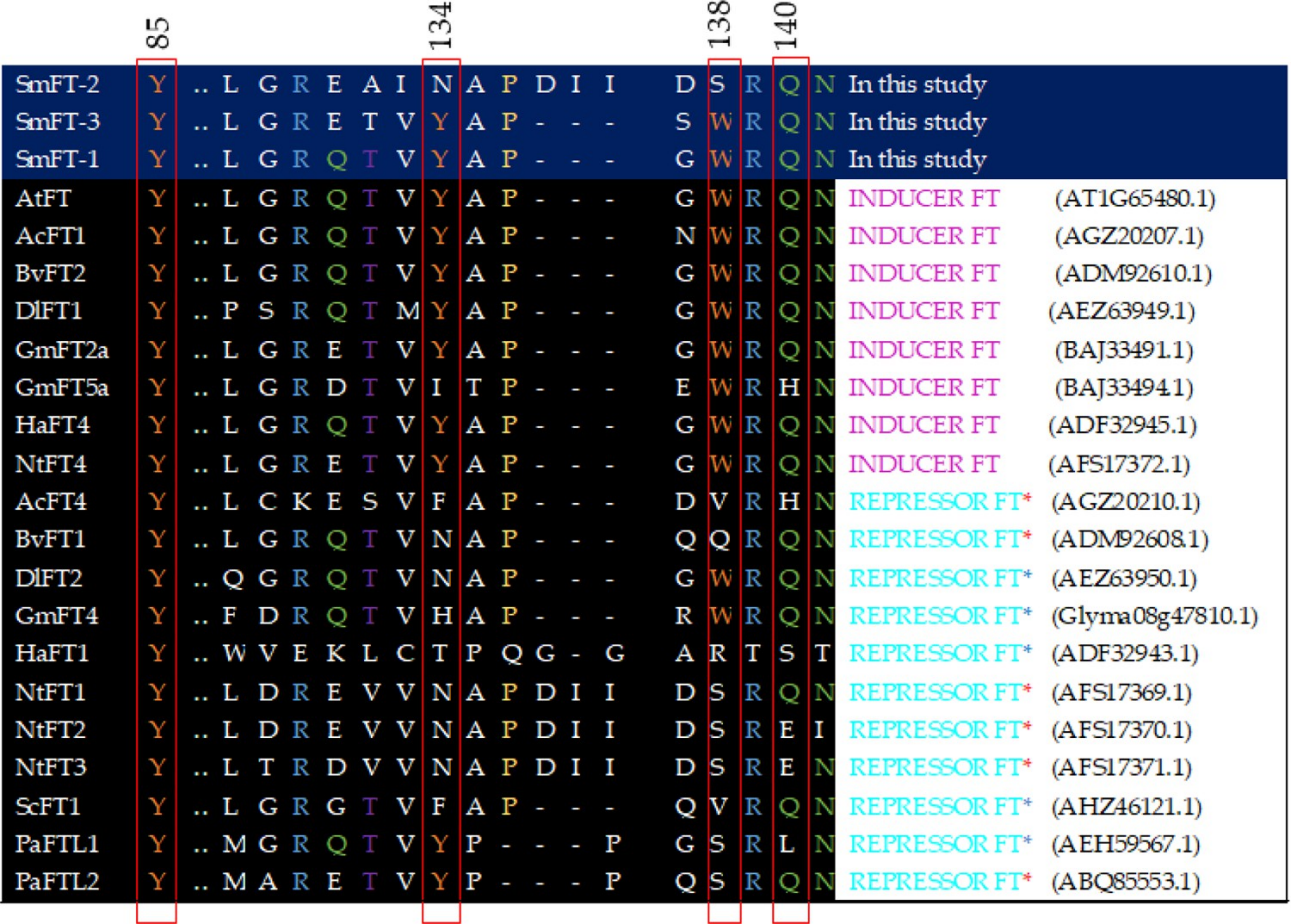
The alignment of the deduced amino acids sequence *of FT/TFL1* gene homologs with inducer *FTs* and repressor *FTs* at the highly-conserved external loop region of exon IV. Amino acid sequence alignment shows conserved position 85 of exon II as well as segment B of exon IV (positions 128–141) from various species such as *Arabidopsis*, onion, sugar beet, longan, soybean, sunflower, tobacco, sugarcane and Norway spruce. Red asterisks represent floral repressor function as characterized in the gene’s native species while blue asterisks represent floral repressor function as characterized in transgenic *Arabidopsis* [1]. Amino acid positions correspond to *Arabidopsis* protein sequence. Numbers in bracket denotes the accession numbers of the sequences.

All four *FT*-like genes were examined in this study. The results revealed that *SmFT*-4 had missing codons in exon IV, including critical amino acids relevant to *FT* gene activities. The deduced amino acid sequences of the remaining three *FT*-like genes (*SmFT*-1, *SmFT*-2 and *SmFT*-3) were aligned to inducer *FT*s and repressor *FT*s from previous published reports, focusing on the conserved external loop region in exon IV. As shown in Fig 4, most inducer *FT*s contains tyrosine (Y) at the 134th position, while repressor *FT*s contains non-tyrosine amino acids at this position (with exceptions being *GmFT5a* for inducer *FT*s and *PaFTL1* and *PaFTL2* for repressor *FT*s). Additionally, all inducer *FT*s carry tryptophan (W) at the 138th position while most repressor *FT*s carries non-tryptophan protein sequence (excluding *DlFT2* and *GmFT4*).

Based on the sequence alignment, both *SmFT*-1 and *SmFT*-3 possess the amino acids, Y85, Y134, W138 and Q140 which are typical of an inducer *FT*. These residues have been well established as factors distinguishing the activator and repressor activities in *FT* [1]. However, *SmFT*-2 deviates from these critical amino acids, as it contains a non-tyrosine amino acid at the 134th position and a non-tryptophan amino acid at the 138th position. This deviation in *SmFT*-2 (Fig 4) is identical to that of the repressor *FT* found in *Nicotiana tabacum* (*NtFT1*). Additionally, the presence of residues E109 and N152, which are important for floral activities [1], were also screened in all the three *FT*-like genes of *S*. *melongena* and were found to be present in their respective positions. Of interest, the upstream region of *SmFT*-1 from the start codon (a putative promoter region) was subjected to a BLASTn survey against the NCBI non-redundant (nr) database. The results revealed the presence of a retrovirus-related polyprotein from transposon RE1 with a length of 2711 bp located at position -1052 to -3763 from the ATG region. This transposon sequence was also found in *SmFT*-1 of all three cultivars.

### Comparison of eggplant and tomato *FT* gene homologs

The *FT* homologs of eggplant (*S*. *melongena*) and tomato (*S*. *lycopersicum*) shared common features as depicted in Table 3. Initially, six *FT* homologs were identified in tomato and referred to as *SlSP3D*, *SlSP6A*, *SlSP5G*, *SlSP5G1*, *SlSP5G2*, and *SlSP5G3* [42]. However, further investigation revealed that these represent only five *FT* genes [43]. *SmFT*-1 was predicted to be a floral promoter without any alterations detected at critical amino acids determining floral transition. One promoter, *SlSP3D*, has been identified in tomato, to date [42]. The changes in the amino acid positions 134, 137 and 138 of *SmFT*-2 and *SlSP5G* suggests that the former plays a repressor role [42].

**Table 3:** Comparison of the *FT* homologs putative features between eggplant and tomato.

Eggplant *FT* homologs	Tomato *FT* homologs	Similarities and differences between the eggplant and tomato *FT*s
*SmFT*-1	*SlSP3D*	Both are floral promoters
*SmFT*-2	*SlSP5G*	a. Both have amino acid changes at 134, 137 and 138^th^ position of *FT* protein [42]. (Positions correspond to Arabidopsis *FT* positions) b. Both have protein residues “APDII” between 134 and 137^th^ positions of *FT* protein [42].
*SmFT*-3	*SlSP5G2*	a. Sm*FT*-3 have changes in amino acids at 137^th^ position whereas SlSP5G2 contain changes at 137 and 138^th^ positions [42]. b. Both do not carry any additional residues between 134 and 137^th^ positions of *FT* protein [42].
*SmFT*-4	*SlSP6A*	Both have premature stop codons in their last exons [42].
*SmFT*-5 (Smechr1100306.1/Smechr1100307.1)	*FTL1* (*SlSP5G1*/*SlSP5G3*)	*SmFT*-5 represented by two separate coding sequences while both *SlSP5G1* & *SlSP5G3* (*FTL1*) found to encode one single protein [43].

Additionally, *SmFT*-4 and *SlSP6A* contained a premature stop codon in their last exons. Most notably, the screening of coding sequences from the *S*. *melongena*-HQ genome resulted in the identification of two separate partial coding sequences for *SmFT*-5, which was similar to *FTL1* in tomato [43].

### *FT/TFL1* gene variants discovered across three different genomic resources

The comparison of each allele of the *FT*/*TFL1* gene homologs mined across three different genome assemblies had uncovered variants in the coding regions as indicated in Table 4. The variants were identified in *SmFT*-2 and *SmCEN*-4. The *SmFT*-2 allele sequence obtained from *S*. *melongena*-HQ differed from the consensus CDS at two different amino acid positions. Likewise, the variant obtained for *SmCEN*-4 had two variations in the protein sequence in comparison to the consensus CDS. According to SIFT prediction, both the variations detected in *SmFT*-2 were targeted to affect protein function, while variations detected for *SmCEN*-4 were tolerated.

**Table 4:** Details of the alterations of amino acids of *Variant1* and *Variant2* from their corresponding deduced protein residues of consensus sequences.

Variants	Altered amino acid	Genomic location	Deduced Protein Position	SIFT prediction	Common residue in *A*. *thaliana*
*SmFT*-2	R to C	Exon IV	127	Not tolerated	R
(*Variant1*)	S to P	Exon IV	156	Not tolerated* (P to S)	P
*SmCEN*-4	I to V	Exon I	19	Tolerated	V
(*Variant2*)	V to A	Exon I	52	Tolerated	T

Note: R, C, S, P, I, V and A denotes amino acids of Arginine, Cysteine, Serine, Proline, Isoleucine, Valine, and Alanine, respectively.

### Allele mining of *SmMFT*-1, *SmMFT*-2, *SmCEN*-1, *SmCEN*-2, *SmCEN*-4 and *SmTFL1* across different cultivars using Pacbio’s long-range amplicon sequencing

In order to dissect the allelic sequence variations in the gene pool of *FT*/*TFL1* homologs of eggplant, Pacbio RS II long-range amplicon sequencing was employed. Here, the sequencing of the full-length open reading frames inclusive of both exons and introns was conducted. The sequencing of *SmMFT*-1, *SmMFT*-2, *SmCEN*-1, SmC*EN*-2, *SmCEN*-4 and *SmTFL1* across four cultivars namely Surya, EP-47 Annamalai, Pant Samrat and Arka Nidhi led to the identification of two genotypes among the genes except for *SmCEN*-4 and *SmTFL1*.

Differences between the genotypes comprised of base substitutions and deletions of single base pairs as well as a stretch of multiple base pairs, as shown in Table 5. The variations fell in the non-coding regions with a small number of them in the genic regions, specifically in *SmMFT*-2. The comparison between the coding regions of these gene homologs and the consensus sequences mined from genome assemblies showed that they were identical and notably *SmCEN*-4 of these cultivars were found to be identical to *Variant 2*. The gene sequences were deposited in NCBI Genbank with following accessions: *TFL1* (OQ025058), *CEN*-1_*allele*1 (OQ025059), *CEN*-1_*allele*2 (OQ025060), *CEN*-2_*allele*1 (OQ025061), *CEN*-2_*allele*2 (OQ025062), *CEN*-4 (OQ025057), *MFT*-1_*allele*1 (OQ025063), *MFT*_1_*allele*2 (OQ025064), *MFT*-2_*allele*1 (OQ025065), *MFT*-2_*allele*2 (OQ025066).

**Table 5:** Genotypes of *TFL*-like and *MFT*-like genes of Surya, EP-47 Annamalai, Pant Samrat, Arka Nidhi as derived from Pacbio RSII amplicon sequencing and the variations between them.

Gene	Alleles	Length (bp)	Cultivars	Number of variations (Introns)	Number of variations (Exons)
*SmTFL*1	*SmTFL1_allele*1	2620	A, B, C, D	-	-
*SmCEN*-1	*SmCEN*-1_*allele*1	1027	B, C, D	2 substitutions and a 12 bp-deletion	-
	*SmCEN*-1_*allele*2	1015	A, B		
*SmCEN*-2	*SmCEN*-2_*allele*1	2596	A, B	6 substitutions	-
	*SmCEN*-2_*allele*2	2596	B, C, D		
*SmCEN*-4	*SmCEN*-4_*allele*1	1488	A, B, C, D	-	-
*SmMFT*-1	*SmMFT*-1_*allele*1	2134	A, B, C	1 substitution	-
	*SmMFT*-1_*allele*2	2134	D		
*SmMFT*-2	*SmMFT*-2_*allele*1	981	A, B, C, D	2 substitutions, a 1 bp-deletion and a 4 bp-deletion	4 non-synonymous variations
	*SmMFT*-2_*allele*2	976	C		

Note: The cultivars denoted by A, B, C, and D correspond to the cultivars Surya, EP-47 Annamalai, Pant Samrat, and Arka Nidhi, respectively.

Unlike other sequenced genes, *SmMFT*-2 displayed more than 90× of amplicon coverage with ~ 460–490 subread coverage which were distributed across almost maximum number of samples, as shown in Table 6. Since higher coverages offer greater possibilities to unveil heterozygosity [44], the samples were screened for such occurrences. There were altogether two alleles detected for the gene. Among them, *SmMFT*-2_*allele*1 was found in all cultivars examined, including Pant Samrat. Interestingly, in Pant Samrat, the *SmMFT*-2_*allele*1 was discovered along with *SmMFT*-2_*allele*2 which was detected in a 1:1 ratio. We verified this with sequences of mutant populations of Pant Samrat (S4 Table).

**Table 6:** Screening for heterozygous *SmMFT*-2 genes through Pacbio RSII amplicon sequencing across Surya (VI045276), EP-47 Annamalai (VI047336), Pant Samrat (VI045550) and Arka Nidhi (VI045274) cultivars.

Accession	Sample Identity	Subread Coverage	Amplicon Coverage	Coverage Ratio	Allele Type
VI045276	Ry_C1	478	169	1	*MFT-*2-*allele*1
	Ry_C2	426	140	1	*MFT-*2-*allele*1
	Ry_C7	482	186	1	*MFT-*2-*allele*1
VI047336	An_C10	464	139	1	*MFT-*2-*allele*1
	An_C13	349	169	1	*MFT-*2-*allele*1
VI045550	Sa_C7	482	91	1	*MFT-*2-*allele*1
	Sa_C8	462	159	1	*MFT-*2-*allele*1
	Sa_C9*	219	58	0.5	*MFT-*2-*allele*1
	Sa_C9*	260	61	0.5	*MFT-*2-*allele*2
VI045274	Ni_C3	488	98	1	*MFT-*2-*allele*1
	Ni_C8	477	160	1	*MFT-*2-*allele*1
	Ni_C1	475	134	1	*MFT-*2-*allele*1

* Two alleles were detected in this heterozygous locus.

The gene coverages of the *SmMFT*-2 in the mutant population were also similar to the control samples detailed in Table 6 and there were 10 samples (Surya), 7 samples (EP-47 Annamalai), 8 samples (Pant Samrat) and 2 samples (Arka Nidhi) sequenced for this homolog. Based on this additional analysis, *SmMFT*-2_*allele*1 was observed across all the four samples. Meanwhile, *SmMFT*-2_*allele*2 was not found in Surya, EP-47 Annamalai and Arka Nidhi but was present in one half of the samples of Pant Samrat in the heterozygous form, similar to earlier findings.

Both *SmMFT-*2_*allele*1 and *SmMFT-*2_*allele*2 differ from each other by two substitutions, a 1 bp-deletion and a 4 bp-deletion in the intronic regions and four non-synonymous mutations in the coding regions. Based on the full-length coding sequence, the nucleotide variations encompass positions 61, 277, 350 and 367, as shown in Table 7. Variations at positions 61, 277 and 350 were predicted to impact protein functions (SIFT analysis).

**Table 7:** Details of the *SmMFT*-2_*allele*2 nucleotide and the corresponding amino acid variations from *SmMFT*-2_*allele*1.

Nucleotide position	Exon	Substitution	Corresponding amino acid	Deduced Protein Position	SIFT Prediction
61	I	G to T	V to F	21	Not tolerated
277	III	A to C	I to L	93	Not tolerated
350	IV	T to G	I to R	117	Not tolerated
367	IV	G to A	A to T	123	Tolerated

### Comparative study of *MFT*-like gene sequences in *S*. *melongena* with transcripts of its wild relative, *S*. *incanum*

The *MFT*-like genes were mined from the *de novo* assembled transcriptome sequences of *S*. *incanum*, the wild relative of *S*. *melongena*. The coding sequences of *SmMFT*-1 gene had one variation as compared to the *MFT*-1 transcript of *S*. *incanum* (hereafter referred to as *SiMFT*-1). The corresponding amino acid variation from Threonine (T) to Serine (S) was predicted to be tolerated (SIFT analysis).

**Fig 5:**
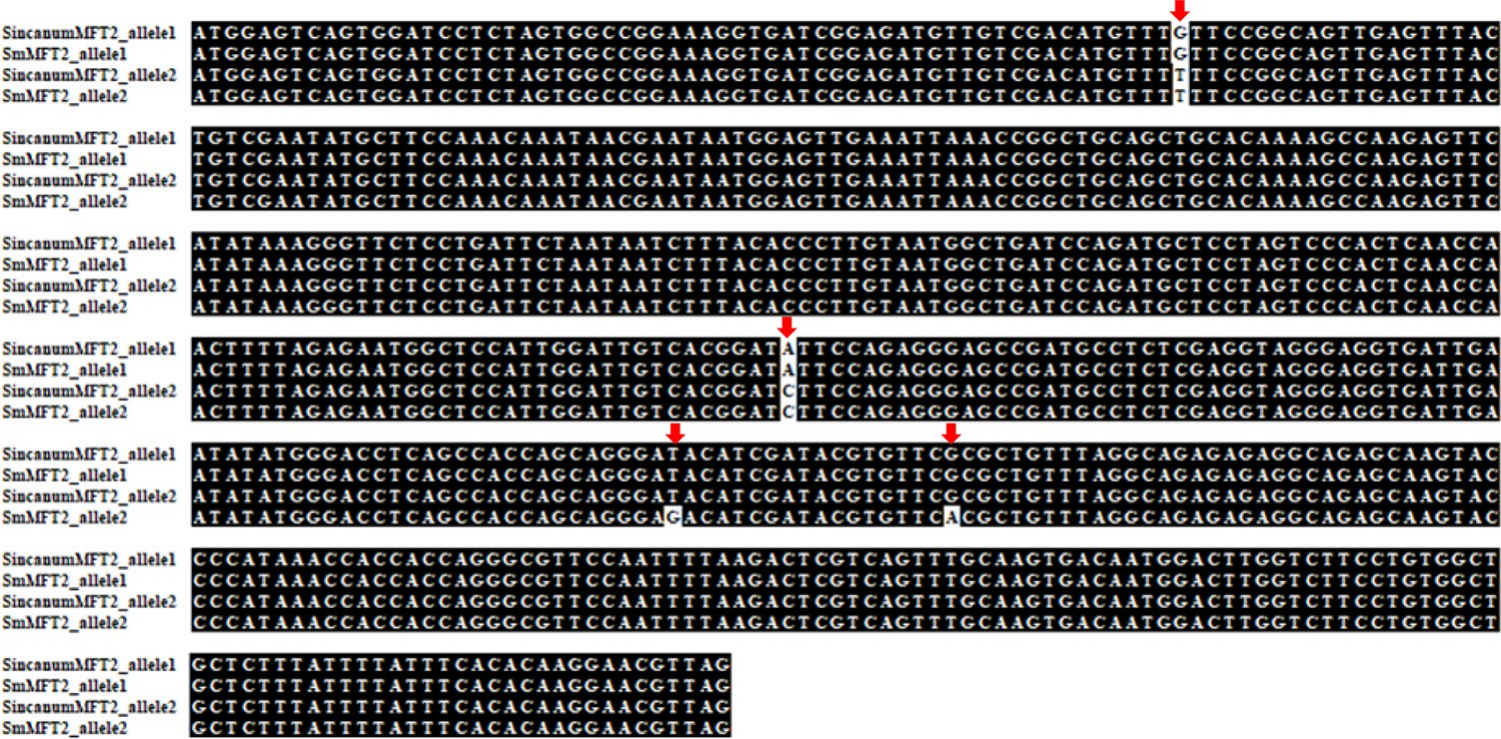
Sequence comparisons among alleles of *S*. *incanum* and the alleles of *S*. *melongena* identified in this study. Red arrows indicate the loci which carry the variations.

Next, the exploration of *MFT*-2 genes in *S*. *incanum* unveiled the homolog to be heterozygous (one of the alleles is termed as *SiMFT*-2_*allele*1 and the other *SiMFT-*2_*allele*2, hereafter). The coding sequences of these alleles differed in positions 67 and 277 (the positions referred here as in the coding sequence). Interestingly, as shown in Fig 5, the genic regions of *SiMFT*-2_*allele*1 and *SmMFT*-2_*allele*1 were identical. Meanwhile, coding regions of *SiMFT*-1_*allele*2 and *SmMFT*-2_*allele*2 were identical including at the positions of 67 and 277 except that *SmMFT*-2_*allele*2 carries two additional variations at positions, 350 and 367 in exon IV.

## Discussion

In this study, the availability of multiple genome assemblies of eggplant provided a platform to mine sequences of *FT*/*TFL1* gene homologs with high confidence. This was achieved by comparing sequences from different resources to derive highly identical versions. Altogether, a total of 12 members of the gene family have been uncovered and their putative genomic organizations were found to be similar to those in *Arabidopsis*. While the number of *TFL*-like and *MFT*-like gene homologs was equal across the genome assemblies, there were differences in the number of *FT* paralogs. These differences may be due to the quality of the genome assemblies. The SME_r2.5.1 [35] was highly fragmented, from which only two *FT* paralogs were obtained, while the chromosome level genome assemblies of *S*. *melongena*-67/3 [36] and S. *melongena*-HQ [37] identified four and five *FT* paralogs, respectively.

The presence of five *FT* paralogs in eggplant is in agreement with the findings of duplication and divergence of this gene cluster in various botanical families, including *Solanaceae* [45, 46] *Brassicaceae* [47] and *Salicaceae* [48], whereas single genes have been reported in species such as grapevines, apple trees and citrus. Additionally, eggplant contains two *MFT*-like genes which are similar to other *Solanum* species like tomato, but in contrast to other dicots like *Arabidopsis*, which is represented by a single gene [49]. Besides, the presence of more than one *MFT*-like gene has also been reported in several monocot genomes, for instances, two genes in rice and three genes in maize. Furthermore, it is interesting to note that eggplant with its 12 *FT*/*TFL1* genes share identical number of the genes with its congener, tomato [42, 43]. This suggests that the genome mining of *FT/TFL1* genes in eggplant has likely resulted in a complete or near-complete collection of the homologs.

### Characteristics of *FT/TFL1* gene homologs in *S*. *melongena*

The sequence analysis of *FT*-like genes of *S*. *melongena* revealed structural and putative functional divergence among individual paralogs, as predicted through protein sequence alignment with previously characterized *FT* promoters and repressors. Screening of residues Tyr-134 Trp-138 codons, commonly present in floral activators [1], revealed that *SmFT*-1 matched the predicted promotor, suggesting its role in promoting floral induction as based on *Arabidopsis*.

*SmFT*-2, however, exhibited variations in these critical regions, with Y134N and W138S substitutions. This is consistent with the functional shift observed in the sugar beet *FT* ortholog *BvFT2*, where three mutations in the external loop region (residues Tyr-134, Gly-137 and Trp-138) were associated to conversion of *BvFT2* into a floral repressor. To investigate the effect of individual changes in these residues, mutations were introduced in these positions in *Arabidopsis FT*. Introduction of mutations such as G137A, G137W, G137E (which is similar to G137Q) and G137R did not impart any repressive activity on the *Arabidopsis FT*. Furthermore, the introduction of point mutations at Tyr-134 and Trp-138 was able to convert the *FT* into a *TFL1*-like molecule and it was discovered that manipulating either of the residues was sufficient to confer floral repressive activity on the *FT* [50]. Similar accounts of functional shifts were also shared by *FT* orthologs of various species. Interestingly, the amino acid variations observed in *SmFT*-2 in these conserved positions were identical to the floral repressors of tobacco *NtFT1*, *NTFT2* and *NtFT3* [1] as well as that of tomato (*SlSP5G*) [42], as previously documented. This is indicative that floral repressing activity was acquired by *SmFT-2*, through evolution of the *FT* clade. Finally, *SmFT*-3 has shown variations at Gly-137 position. However, further functional validation is required to elucidate the impact of this positional change in eggplant. In addition to the residues described, mutations at other amino acids such as Tyr-85, Glu-109, Gln-140 and Asn-152 have been reported to impact the functional specificity of *FT* and *TFL1* in Arabidopsis [50]. These residues were found to be invariant in these genes examined. The mutations at Tyr-134 and Trp-138 indicate successive evolutionary changes of the *FT* clade after the divergence of *FT*-like and *TFL1*-like genes [1].

We also identified the presence of a transposable element in the putative promoter of *SmFT*-1, located in the upstream region of the homolog (-1052 to -3763 from the start codon). Transposable elements constitute a significant portion of plant DNA and their effect on gene expression can vary depending on their location [51]. Further investigation is necessary to determine whether this specific element affects the expression of the gene. Additionally, one of the *SmFT* paralogs, *SmFT*-4, contains a premature stop codon in exon IV. Truncated codons in the *FT* gene have been found in tomato. An *FT* homolog in tomato, *SlSP6A*, had a premature stop codon in exon IV and its expression has not been detected in various organs, leading to its prediction as a pseudogene [42]. In addition to the *FT* genes, analysis of the *TFL*-like and *MFT*-like gene sequences in eggplant showed that they were invariant at the known critical amino acids.

Variations in *FT* paralogs reflect a strategy of the crop to precisely time flowering in response to diverse external and internal stimuli [42]. For example, in sugar beet, *BvFT1* is a floral repressor that prevents flowering under short-day conditions and before vernalization by repressing *BvFT2* expression [52]. Similarly, some Solanaceous crops have undergone gene duplication and divergence in *FT* homologs to fine-tune flowering initiation in response to environmental stimuli. In tobacco, *NtFT4* promotes flowering whereas *NtFT1*, *NtFT2* and *NtFT3* are floral repressors, all of which are expressed during short day conditions [10]. Recently, a novel *FT* gene, *NtFT5*, was discovered to be expressed regardless of the day length, suggesting a regulatory role under both long-day and short-day conditions [53]. In tomato, there are three *FT* orthologs that act as floral repressors. One of them is triggered by long days, while the other two are triggered by short days. Moreover, an *FT* homolog of tomato, known as *SFT*/*SP3D*, is a floral promoter whose expression is insensitive to photoperiod [42]. As such, the divergence of *FT* paralogs in eggplant is likely to confer differential activities influencing floral transitions in response to environmental cues. The identification of gene homologs provides additional avenues to further our understanding of flowering mechanisms in eggplant. The genome mining indicates the presence of variants such as *Variant1* of *SmFT*-2 and *Variant2* of *SmCEN*-4. Substitutions found in *Variant1* were predicted to affect protein function (SIFT analysis) while the changes in *Variant2* were possibly tolerated. Taken together, our findings support the postulation that individual eggplant cultivars carry gene variants which are absent in the reference genome which can be unveiled through resequencing of various accessions and such variants have been expected to influence phenotypic traits [36].

### Comparison of *FT* homologs between eggplant and tomato

Five *FT* homologs were identified from the genomes of eggplant and tomato [42, 43]. We found basic similarities between the *FT* homologs of both *Solanum* species, as described in Table 3. Interestingly, we obtained two partial coding sequences from the genomic resources of eggplant that corresponded to a single gene, *SmFT*-5. We retrieved the upstream and downstream sequences of these partial sequences from the corresponding genomic scaffolds and subjected them to Fgenesh gene structure prediction (S2 Fig). Fgenesh predicted two partial sequences. We expect that some mutations may have caused inaccuracies in the predicted gene structure, although it is also possible that the genome assembly process introduced sequence variations. In tomato, a comparable circumstance was encountered during the genome-wide identification of *FT/TFL1* gene homologs, where six *FT* gene sequences were initially identified, but two were later found to correspond to a single gene, called *Flowering Locus T*-*Like 1* (*FTL1*) [42, 43]. A 2 bp deletion in the *FTL1* gene had resulted in the premature termination of the protein, producing a fragmentary PEBP domain [43]. These findings from another *Solanum* species suggest that further investigations are necessary to verify the gene structure of eggplant *SmFT*-5.

### Utilization of long-range targeted sequencing of Pacbio in the allele mining of *FT/TFL1* gene homologs across different cultivars

Since allele mining is an efficient way to gather gene variants and study their implications in agricultural adaptations [54], we extended our search for additional variants among the commercial cultivars of Surya, EP-47 Annamalai, Pant Samrat and Arka Nidhi. We used Pacbio RSII long-range targeted amplicon sequencing to discover different genotypes in *SmMFT*-1, *SmMFT*-2, *SmCEN*-1, *SmCEN*-2, *SmCEN*-4 and *SmTFL*-1. We found that, except for *SmCEN*-4 and *SmTFL*-1, each of the genes showed presence of two different genotypes among the cultivars. Screening for variations between the alleles of each gene revealed that most variations fell in intronic regions, while a few resided in the exonic region, which is consistent with the high conservation of protein coding sequence [55].

We used a single barcode type for the entire gene homologs (~1kbp to ~3kbp in lengths) extracted from an individual plant, despite high sequence similarities between these members of a multi-gene family. This was possible due to the long-range sequencing property of the Pacbio platform which negates the need to fragment long sequences and reconstruct them back during assembly [56]. However, as the amplicons sequenced were of unequal size distributions, the gene coverages obtained were also uneven. Nonetheless, some plant samples showed the presence of heterozygous loci which contain both alleles. Here, the long-read amplicon sequencing provided a straightforward remedy for variant phasing [57].

Sequencing of the gene homolog, *SmMFT*-2 however resulted in almost equal and highest range of coverages, as per this study. Some members of the Pant Samrat cultivar were shown to be heterozygous at the locus (i.e. *SmMFT*-2_*allele*1 and *SmMFT*-2_*allele*2). However, *SmMFT*-2_*allele*2 was absent in any other cultivars examined, as only *SmMFT*-2_*allele*1 was detected among them. Comparison of the two alleles indicates the presence of four non-synonymous mutations, three of which (at positions 61, 277 and 350) could impacts protein function, as predicted by the SIFT analysis.

### Comparative study of *SmMFT* gene sequences with transcripts of its wild relative, *S*. *incanum*

*MFT* gene homologs are considered to be the ancestors of *FT*/*TFL1* gene sequences. However, its function is not well understood. In addition to their redundant floral inductive function, these homologs also play important roles in seed dormancy and germination, in line with their seed specific expressions [58]. In *Arabidopsis*, *MFT* has been shown to increase dormancy during seed development stage, while promoting germination in after-ripened seeds imbibed with exogenous ABA. In contrast, *Triticum aestivum MFT* (*TaMFT*) positively regulates seed dormancy while inhibiting seed germination [16]. In *S*. *melongena*, seed dormancy is considered to be a trait that has undergone human selection since it is rarely related in this species [59]. Nevertheless, the trait is commonly observed in wild-type *Solanum* species [60]. *S*. *incanum*, a wild ancestor of *S*. *melongena*, has been reported to display a slow and low germination rate, taking about 30 days to reach 15 to 50% germination [61]. Therefore, a comparative genetic analysis has been undertaken to determine coding sequence differences that could possibly exist between *S*. *incanum* and the cultivars examined in this study.

Through the mining of *MFT*-like genes using *S*. *incanum* transcriptome, we identified an allele related to *SmMFT*-1 homolog. We found one non-synonymous nucleotide variation between *SmMFT*-1 and the corresponding allele detected in the *S*. *incanum* transcript. Based on the results of the SIFT analysis, the corresponding amino acid variation was predicted to be tolerated.

With regard to the *MFT*-2 gene, *S*. *incanum* showed the presence of heterozygous alleles, which were named as *SiMFT*-2_*allele*1 and *SiMFT*-2_*allele*2, respectively. Similar to the cultivar Pant Samrat, *SiMFT*-2_*allele*1 and *SiMFT*-2_*allele*2 differed at positions 61 and 277 (referring to the coding sequences in line with *SmMFT*-2 genes). *SiMFT*-2_*allele*1 was detected and found to be identical across cultivars, Surya, EP-47 Annamalai, Pant Samrat and Arka Nidhi. All the cultivars showed homozygosity for the allele, except for Pant Samrat. The other allele of *S*. *incanum* (*SiMFT*-2_*allele*2) was distributed in some members of Pant Samrat, where *SmMFT*-2_*allele*2 shared similar nucleotides at positions 61 and 277. However, the latter carried two additional mutations at positions 350 and 367. The absence of *SmMFT*-2_*allele*2 genotype in most cultivars could be responsible for the discrepancies in seed traits between wild type and most cultivated species. However, these findings need to be further supported by empirical data.

## Conclusions

The functional interpretations of *FT*/*TFL1* gene homologs through computational approaches indicate the divergence of *FT* paralogs. Similar functional alterations have been demonstrated to modulate floral regulation in various species, including Solanaceous crops like tomato [42] and tobacco [10]. Therefore, such changes in *FT* paralogs in eggplant could be implicated on the crop’s differential responses to environmental signals. This study has also uncovered unique variations in the *MFT*-2 gene sequences across different cultivars, as well as in comparison to the wild relative, *S*. *incanum*. These variations suggest a possible role of these alleles in regulating seed dormancy and germination. The hypotheses derived from this study add to the fundamental points to direct future functional validations pertaining to floral regulation, seed dormancy and germination in eggplant.

## Supporting information

S1 Fig Comparison of *MFT*-2 gene mined from Sme_r2.5.1 and the corresponding transcript of *S*. *melongena* (Ramnagar Giant). (DOCX)

S2 Fig The coding sequence (CDS) of *SmFT*-5 as predicted using Fgenesh gene prediction tool. (DOCX)

S1 Table Sequences of primers used in the first round of PCR. (DOCX)

S2 Table Sequences of primers used in the second round of PCR. (DOCX)

S3 Table Primer combinations used in the second round of PCR based on cultivars. (DOCX)

S4 Table Distribution of unmutated SmMFT-2 alleles in the mutant populations of cultivars, Surya, EP-47 Annamalai, Pant Samrat and Arka Nidhi. (DOCX)

## References

[1] Wickland DP, Hanzawa Y. The *FLOWERING LOCUS T/TERMINAL FLOWER 1* gene family: functional evolution and molecular mechanisms. Mol Plant. 2015; 8: 983–997. doi: 10.1016/j.molp.2015.01.007 25598141

[2] Park SJ, Jiang K, Tal L, Yichie Y, Gar O, Zamir D, et al. Optimization of crop productivity in tomato using induced mutations in the florigen pathway. Nat Genet. 2014; 46: 1337–1342. doi: 10.1038/ng.3131 25362485

[3] Higuchi Y. Florigen and anti-florigen: flowering regulation in horticultural crops. Breed Sci. 2018; 68: 109–118. doi: 10.1270/jsbbs.17084 29681753 PMC5903977

[4] Moraes TS, Dornelas MC, Martinelli AP. *FT/TFL1*: calibrating plant architecture. Front Plant Sci. 2019; 10: 97. doi: 10.3389/fpls.2019.00097 30815003 PMC6381015

[5] Li C, Fu Q, Niu L, Luo L, Chen J, Xu ZF. Three *TFL1* homologues regulate floral initiation in the biofuel plant *Jatropha curcas*. Sci Rep. 2017; 7: 1–9. doi: 10.1038/srep43090 28225036 PMC5320528

[6] Lee JH, Lee JS, Ahn JH. Ambient temperature signaling in plants: an emerging field in the regulation of flowering time. J Plant Biol. 2008; 51: 321–326. 10.1007/BF03036133

[7] Srikanth A, Schmid M. Regulation of flowering time: all roads lead to Rome. Cell Mol Life Sci. 2011; 68: 2013–2037. doi: 10.1007/s00018-011-0673-y 21611891 PMC11115107

[8] Liu L, Ou C, Chen S, Shen Q, Liu B, Li M, et al. The response of *COL* and *FT* homologues to photoperiodic regulation in carrot (*Daucus carota* L.). Sci Rep. 2020; 10: 1–13. doi: 10.1038/s41598-020-66807-y 32561786 PMC7305175

[9] Zhang M, Li P, Yan X, Wang J, Cheng T, Zhang Q. Genome-wide characterization of PEBP family genes in nine *Rosaceae* tree species and their expression analysis in *P*. *mume*. BMC Ecol. 2021; 21: 1–23. doi: 10.1186/s12862-021-01762-4 33622244 PMC7901119

[10] Harig L, Beinecke FA, Oltmanns J, Muth J, Müller O, Rüping B, et al. Proteins from the *FLOWERING LOCUS T*-like subclade of the PEBP family act antagonistically to regulate floral initiation in tobacco. Plant J. 2012; 72: 908–921. doi: 10.1111/j.1365-313X.2012.05125.x 22889438

[11] Zhu Y, Klasfeld S, Jeong CW, Jin R, Goto K, Yamaguchi N, et al. *TERMINAL FLOWER 1-FD* complex target genes and competition with *FLOWERING LOCUS T*. Nat Commun. 2020; 11: 1–12. doi: 10.1038/s41467-020-18782-1 33046692 PMC7550357

[12] Kim G, Rim Y, Cho H, Hyun TK. Identification and Functional Characterization of *FLOWERING LOCUS T* in *Platycodon grandiflorus*. Plants. 2022; 11: 325. doi: 10.3390/plants11030325 35161306 PMC8840131

[13] Yamaguchi A, Kobayashi Y, Goto K, Abe M, Araki T. *TWIN SISTER OF FT (TSF)* acts as a floral pathway integrator redundantly with *FT*. Plant Cell Physiol. 2005; 46: 1175–1189. doi: 10.1093/pcp/pci151 15951566

[14] Yoo SY, Kardailsky I, Lee JS, Weigel D, Ahn JH. Acceleration of flowering by overexpression of *MFT*. Mol Cells. 2004; 17: 95–101. 15055534

[15] Igasaki T, Watanabe Y, Nishiguchi M, Kotoda N. The *flowering locus t/terminal flower 1* family in *Lombardy poplar*. Plant Cell Physiol. 2008; 49: 291–300. doi: 10.1093/pcp/pcn010 18203732

[16] Li Q, Fan C, Zhang X, Wang X, Wu F, Hu R, et al. Identification of a soybean *MOTHER OF FT AND TFL1* homolog involved in regulation of seed germination. PLoS One. 2014; 9: e99642. 10.1371/journal.pone.0099642 24932489 PMC4059689

[17] Li JX, Hou XJ, Zhu J, Zhou JJ, Huang HB, Yue JQ, et al. Identification of genes associated with lemon floral transition and flower development during floral inductive water deficits: a hypothetical model. Front Plant Sci. 2017; 8: 1013. doi: 10.3389/fpls.2017.01013 28659956 PMC5468436

[18] Karlgren A, Gyllenstrand N, Källman T, Sundström JF, Moore D, Lascoux M, et al. Evolution of the PEBP gene family in plants: functional diversification in seed plant evolution. Plant Physiol. 2011; 156: 1967–1977. doi: 10.1104/pp.111.176206 21642442 PMC3149940

[19] Li R, Wang A, Sun S, Liang S, Wang X, Ye Q, et al. Functional characterization of *FT* and *MFT* ortholog genes in orchid (*Dendrobium nobile* Lindl) that regulate the vegetative to reproductive transition in *Arabidopsis*. Plant Cell Tissue Organ Cult. 2012; 111: 143–151. 10.1007/s11240-012-0178-x

[20] Bi Z, Li X, Huang H, Hua Y. Identification, functional study, and promoter analysis of *HbMFT1*, a homolog of *MFT* from rubber tree (*Hevea brasiliensis*). Int J Mol Sci. 2016; 17: 247. doi: 10.3390/ijms17030247 26950112 PMC4813128

[21] Vaistij FE, Barros-Galvão T, Cole AF, Gilday AD, He Z, Li Y, et al. *MOTHER-OF-FT-AND-TFL1* represses seed germination under far-red light by modulating phytohormone responses in *Arabidopsis thaliana*. PNAS. 2018; 115: 8442–8447. doi: 10.1073/pnas.1806460115 30061395 PMC6099910

[22] Nakamura S, Abe F, Kawahigashi H, Nakazono K, Tagiri A, Matsumoto T, et al. A wheat homolog of *MOTHER OF FT AND TFL1* acts in the regulation of germination. Plant Cell. 2011; 23: 3215–3229. 10.1105/tpc.111.088492 21896881 PMC3203438

[23] Li C, Luo L, Fu Q, Niu L, Xu ZF. Identification and characterization of the *FT/TFL1* gene family in the biofuel plant *Jatropha curcas*. Plant Mol Biol Rep. 2015; 33: 326–333. 10.1007/s11105-014-0747-8

[24] Ryu JY, Lee HJ, Seo PJ, Jung JH, Ahn JH, Park CM. The *Arabidopsis* floral repressor *BFT* delays flowering by competing with *FT* for *FD* binding under high salinity. Mol Plant. 2014; 7: 377–387. doi: 10.1093/mp/sst114 23935007

[25] Navarro C, Abelenda JA, Cruz-Oró E, Cuéllar CA, Tamaki S, Silva J, et al. Control of flowering and storage organ formation in potato by *FLOWERING LOCUS T*. Nature. 2011; 478: 119–22. doi: 10.1038/nature10582 21947007

[26] Shalit A, Rozman A, Goldshmidt A, Alvarez JP, Bowman JL, Eshed Y, et al. The flowering hormone florigen functions as a general systemic regulator of growth and termination. PNAS. 2009; 106: 8392–7. doi: 10.1073/pnas.0810810106 19416824 PMC2688896

[27] Kinoshita T, Ono N, Hayashi Y, Morimoto S, Nakamura S, et al. *FLOWERING LOCUS T* regulates stomatal opening. Curr Biol. 2011; 21: 1232–8. doi: 10.1016/j.nmd.2011.04.012 21737277

[28] Lee R, Baldwin S, Kenel F, McCallum J, Macknight R. *FLOWERING LOCUS T* genes control onion bulb formation and flowering. Nat Commun. 2013; 4: 2884. doi: 10.1177/0333102413515340 24300952

[29] Danilevskaya ON, Meng X, McGonigle B, Muszynski MG. Beyond flowering time: pleiotropic function of the maize flowering hormone florigen. Plant Signal Behav. 2011; 6: 1267–70. doi: 10.4161/psb.6.9.16423 21847027 PMC3258048

[30] Chapman MA. Introduction: The importance of eggplant. In: The Eggplant Genome. Springer, Cham; 2019. p. 1–10. doi: 10.1007/978-3-319-99208-2_1

[31] FAOSTAT. Food and Agriculture Organization of the United Nations (FAO). Available from: https://www.fao.org/faostat/en/#data/QCL [Accessed 22 January 2023].

[32] Chioti V, Zeliou K, Bakogianni A, Papaioannou C, Biskinis A, Petropoulos C, et al. Nutritional value of eggplant cultivars and association with sequence variation in genes coding for major phenolics. Plants. 2022; 11: 2267. doi: 10.3390/plants11172267 36079649 PMC9460228

[33] Sidhu AS, Bal SS, Behera TK, Rani M. An outlook in hybrid eggplant breeding. J New Seeds. 2004; 6: 15–29. 10.1300/J153v06n02_02

[34] Sękara A, Cebula S, Kunicki E. Cultivated eggplants–origin, breeding objectives and genetic resources, a review. Folia Hortic. 2007; 19: 97–114.

[35] Hirakawa H, Shirasawa K, Miyatake KOJI, Nunome T, Negoro S, Ohyama KIO, et al. Draft genome sequence of eggplant (*Solanum melongena* L.): the representative solanum species indigenous to the old world. DNA Res. 2014; 21: 649–660. doi: 10.1093/dnares/dsu027 25233906 PMC4263298

[36] Barchi L, Rabanus-Wallace MT, Prohens J, Toppino L, Padmarasu S, Portis E, et al. Improved genome assembly and pan-genome provide key insights into eggplant domestication and breeding. Plant J. 2021; 107: 579–596. doi: 10.1111/tpj.15313 33964091 PMC8453987

[37] Wei Q, Wang J, Wang W, Hu T, Hu H, Bao C. A high-quality chromosome-level genome assembly reveals genetics for important traits in eggplant. Hortic Res. 2020; 7: 153. doi: 10.1038/s41438-020-00391-0 33024567 PMC7506008

[38] Kumar S, Stecher G, Li M, Knyaz C, Tamura K. MEGA X: Molecular Evolutionary Genetics Analysis across computer platforms. Mol Biol Evol. 2018; 35: 1547–1549. doi: 10.1093/molbev/msy096 29722887 PMC5967553

[39] Webb DM, Knapp SJ. DNA extraction from a previously recalcitrant plant genus. Plant Mol Biol Rep. 1990; 8: 180–185. 10.1007/BF02669514

[40] Mishra P, Tripathi AN, Kashyap SP, Aamir M, Tiwari KN, Singh VK, et al. In silico mining of WRKY TFs through *Solanum melongena* L. and *Solanum incanum* L. transcriptomes and identification of SiWRKY53 as a source of resistance to bacterial wilt. Plant Gene. 2021; 26: 100278. 10.1016/j.plgene.2021.100278

[41] Heller WP, Ying Z, Davenport TL, Keith LM, Matsumoto TK. Identification of members of the Dimocarpus Longan Flowering Locus T gene family with divergent functions in flowering. Trop Plant Biol. 2014; 7: 19–29. 10.1007/s12042-013-9134-0

[42] Cao K, Cui L, Zhou X, Ye L, Zou Z, Deng S. Four tomato *FLOWERING LOCUS T*-like proteins act antagonistically to regulate floral initiation. Front Plant Sci. 2016; 7: 1213. doi: 10.3389/fpls.2015.01213 26793202 PMC4707262

[43] Song J, Zhang S, Wang X, Sun S, Liu Z, Wang K, et al. Variations in both *FTL1* and *SP5G*, two tomato *FT* paralogs, control day-neutral flowering. Mol Plant. 2020; 13: 939–942. doi: 10.1016/j.molp.2020.05.004 32417308

[44] Bryc K, Patterson N, Reich D. A novel approach to estimating heterozygosity from low-coverage genome sequence. Genetics. 2013; 195: 553–561. doi: 10.1534/genetics.113.154500 23934885 PMC3781980

[45] Carmel-Goren L, Liu YS, Lifschitz E, Zamir D. The *SELF-PRUNING* gene family in tomato. Plant Mol Biol. 2003; 52: 1215–1222. doi: 10.1023/b:plan.0000004333.96451.11 14682620

[46] Lifschitz E, Eviatar T, Rozman A, Shalit A, Goldshmidt A, Amsellem Z, et al. The tomato *FT* ortholog triggers systemic signals that regulate growth and flowering and substitute for diverse environmental stimuli. PNAS. 2006; 103: 6398–6403. doi: 10.1073/pnas.0601620103 16606827 PMC1458889

[47] Kobayashi Y, Kaya H, Goto K, Iwabuchi M, Araki T. A pair of related genes with antagonistic roles in mediating flowering signals. Science. 1999; 286: 1960–1962. doi: 10.1126/science.286.5446.1960 10583960

[48] Brunner AM, Nilsson O. Revisiting tree maturation and floral initiation in the poplar functional genomics era. New Phytol. 2004; 164: 43–51. doi: 10.1111/j.1469-8137.2004.01165.x 33873486

[49] Carmona MJ, Calonje M, Martínez-Zapater JM. The *FT/TFL1* gene family in grapevine. Plant Mol Biol. 2007; 63: 637–650. doi: 10.1007/s11103-006-9113-z 17160562

[50] Ho WWH, Weigel D. Structural features determining flower-promoting activity of *Arabidopsis FLOWERING LOCUS T*. Plant Cell. 2014; 26: 552–564. doi: 10.1105/tpc.113.115220 24532592 PMC3967025

[51] Gramazio P, Yan H, Hasing T, Vilanova S, Prohens J, Bombarely A. Whole-genome resequencing of seven eggplant (*Solanum melongena*) and one wild relative (*S*. *incanum*) accessions provides new insights and breeding tools for eggplant enhancement. Front Plant Sci. 2019; 10: 1220. doi: 10.3389/fpls.2019.01220 31649694 PMC6791922

[52] Pin PA, Benlloch R, Bonnet D, Wremerth-Weich E, Kraft T, Gielen JJ, et al. An antagonistic pair of *FT* homologs mediates the control of flowering time in sugar beet. Science. 2010; 330: 1397–1400. doi: 10.1126/science.1197004 21127254

[53] Schmidt FJ, Zimmermann MM, Wiedmann DR, Lichtenauer S, Grundmann L, Muth J, et al. The major floral promoter *NtFT5* in tobacco (*Nicotiana tabacum*) is a promising target for crop improvement. Front Plant Sci. 2020; 10: 1666. doi: 10.3389/fpls.2019.01666 31998348 PMC6966700

[54] Kumar GR, Sakthivel K, Sundaram RM, Neeraja CN, Balachandran SM, Rani NS, et al. Allele mining in crops: prospects and potentials. Biotechnol Adv. 2010; 28: 451–461. doi: 10.1016/j.biotechadv.2010.02.007 20188810

[55] Panaro MA, Calvello R, Miniero DV, Mitolo V, Cianciulli A. Imaging Intron Evolution. Methods Protoc. 2022; 5: 53. doi: 10.3390/mps5040053 35893579 PMC9326662

[56] Zhang W, Ciclitira P, Messing J. PacBio sequencing of gene families—A case study with wheat gluten genes. Gene. 2014; 533: 541–546. doi: 10.1016/j.gene.2013.10.009 24144842

[57] Charnaud S, Munro JE, Semenec L, Mazhari R, Brewster J, Bourke C, et al. PacBio long-read amplicon sequencing enables scalable high-resolution population allele typing of the complex CYP2D6 locus. Commun Biol. 2022; 5: 1–10. doi: 10.1038/s42003-022-03102-8 35217695 PMC8881578

[58] Yu X, Liu H, Sang N, Li Y, Zhang T, Sun J, et al. Identification of cotton *MOTHER OF FT AND TFL1* homologs, *GhMFT1* and *GhMFT2*, involved in seed germination. PLoS One. 2019; 14: e0215771. 10.1371/journal.pone.0215771 31002698 PMC6474632

[59] Yogeesha HS, Upreti KK, Padmini K, Bhanuprakash K, Murti GSR. Mechanism of seed dormancy in eggplant (*Solanum melongena* L.). Seed Sci Technol. 2006; 34: 319–325. 10.15258/sst.2006.34.2.07

[60] Page AM, Daunay MC, Aubriot X, Chapman MA. Domestication of eggplants: a phenotypic and genomic insight. In: The Eggplant Genome. Springer; 2019:193–212. 10.1007/978-3-319-99208-2_12

[61] Joshua A. Seed germination of *Solanum incanum*: an example of germination problems of tropical vegetable crops. Acta Hortic. 1978; 83: 155–162. 10.17660/ActaHortic.1978.83.20

