# Supplementary material for: Allele mining, amplicon sequencing and computational prediction of Solanum melongena L. FT/TFL1 gene homologs uncovers putative variants associated to seed dormancy and germination
Source: PLoS One. 2023 May 3;18(5):e0285119. doi: 10.1371/journal.pone.0285119 (PMC10156061; doi:10.1371/journal.pone.0285119)
Supplement: S1 Fig — (DOCX) [file pone.0285119.s001.docx]

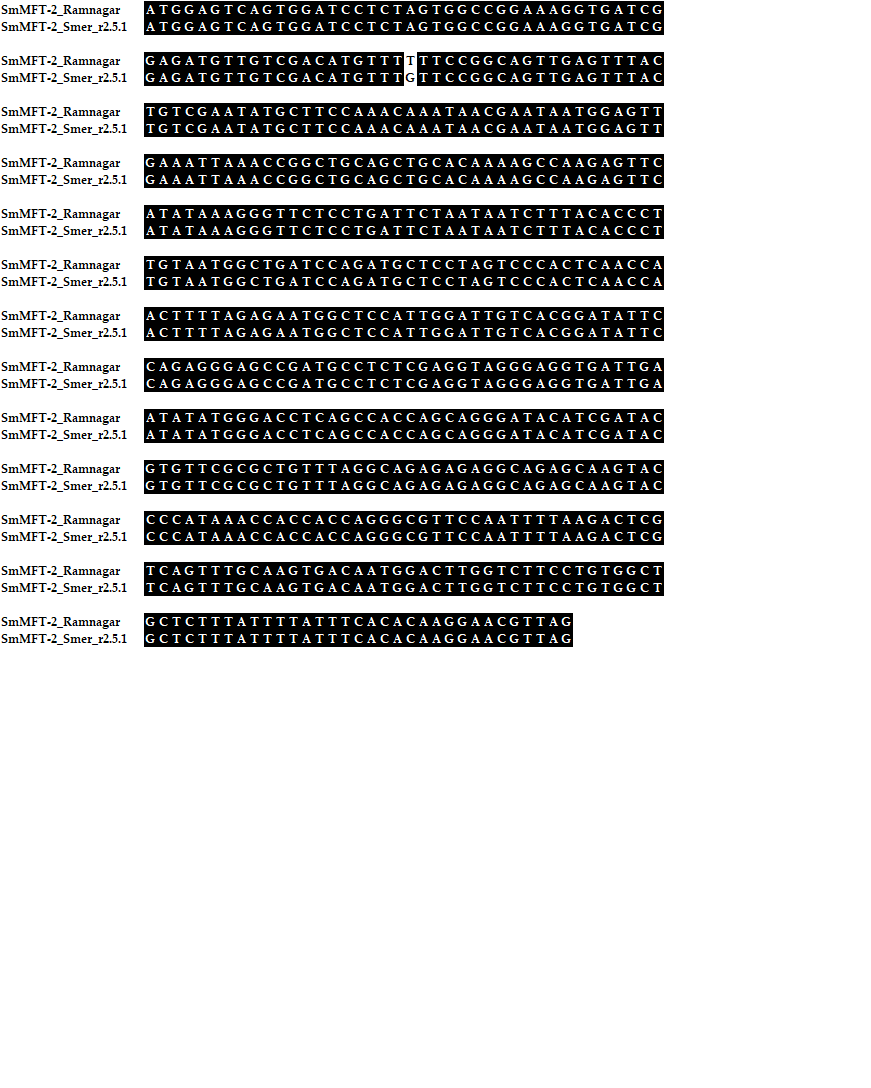


**Figure S1.** Comparison of *MFT*-2 gene mined from Sme_r2.5.1 and the corresponding transcript of *S*. *melongena* (Ramnagar Giant). Black background denotes bases with similarities whereas white background denotes variation.
