# Supplementary material for: Allele mining, amplicon sequencing and computational prediction of Solanum melongena L. FT/TFL1 gene homologs uncovers putative variants associated to seed dormancy and germination
Source: PLoS One. 2023 May 3;18(5):e0285119. doi: 10.1371/journal.pone.0285119 (PMC10156061; doi:10.1371/journal.pone.0285119)
Supplement: S2 Fig — (DOCX) [file pone.0285119.s002.docx]

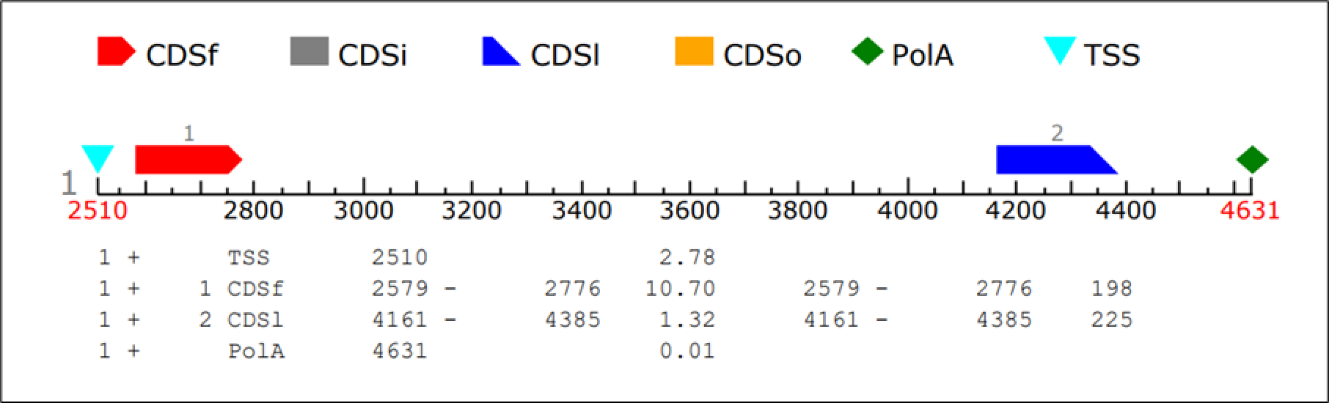


**Figure S2.** Gene structure of the *SmFT*-5 coding sequence (CDS) as predicted by the Fgenesh tool. CDSf indicates the first CDS (which starts with a start codon), CDSi indicates internal exon, CDSo indicates outer exon, and CDSl indicates the last coding sequence (which ends with a stop codon). The Transcription Start Site (TSS) and poly-A tail (PolA) are also indicated in the figure.
