# Supplementary material for: Allele mining, amplicon sequencing and computational prediction of Solanum melongena L. FT/TFL1 gene homologs uncovers putative variants associated to seed dormancy and germination
Source: PLoS One. 2023 May 3;18(5):e0285119. doi: 10.1371/journal.pone.0285119 (PMC10156061; doi:10.1371/journal.pone.0285119)
Supplement: S1 Table — (DOCX) [file pone.0285119.s003.docx]

**Table S1**. Sequences of primers used in the first round of PCR

| **Primer Identity** | **Sequence (direction 5' to 3')** |
| --- | --- |
| CEN1_FOR | /5AmMC6/gcagtcgaacatgtagctgactcaggtcacCTC CTC CTT TGC AAC ATT ATC A |
| CEN2_FOR | /5AmMC6/gcagtcgaacatgtagctgactcaggtcacGGT CCC ATA TTT TTT ATG CCT TC |
| CEN4_FOR | /5AmMC6/gcagtcgaacatgtagctgactcaggtcacCCT TTA ATC TTG AAG CCT GAA CA |
| TFL1_FOR | /5AmMC6/gcagtcgaacatgtagctgactcaggtcacGTA TTT TGC ACT TCT CTT CTC AC |
| MFT1_FOR | /5AmMC6/gcagtcgaacatgtagctgactcaggtcacCGT AAG TGA CCA AAA TCC TTG TAA |
| MFT2_FOR | /5AmMC6/gcagtcgaacatgtagctgactcaggtcacCAG CCA CGT CAG CAT TAA CAT GA |
| CEN1_REV | /5AmMC6/ tggatcacttgtgcaagcatcacatcgtagGTA CAA AGT CGT TGT ACT AGC A |
| CEN2_REV | /5AmMC6/ tggatcacttgtgcaagcatcacatcgtagATG GAT CAA TCA AAC GCT ACT AAG |
| CEN3_REV | /5AmMC6/ tggatcacttgtgcaagcatcacatcgtagGGA CCA AAA TAT TGA CGA CAA AG |
| CEN4_REV | /5AmMC6/ tggatcacttgtgcaagcatcacatcgtagCTA CAT GAT GCA TGA ACA TGA CA |
| TFL1_REV | /5AmMC6/ tggatcacttgtgcaagcatcacatcgtagGAA GAG AGT AAA CAA CAC TAA CC |
| MFT1_REV | /5AmMC6/ tggatcacttgtgcaagcatcacatcgtagGTG CCA AAA CAG AAA ACA CAC AA |
| MFT2_REV | /5AmMC6/ tggatcacttgtgcaagcatcacatcgtagACG AGT CGT GAC ATG AAA CAG TA |
