# Supplementary material for: Allele mining, amplicon sequencing and computational prediction of Solanum melongena L. FT/TFL1 gene homologs uncovers putative variants associated to seed dormancy and germination
Source: PLoS One. 2023 May 3;18(5):e0285119. doi: 10.1371/journal.pone.0285119 (PMC10156061; doi:10.1371/journal.pone.0285119)
Supplement: S2 Table — (DOCX) [file pone.0285119.s004.docx]

**Table S2**. Sequences of primers used in the second round of PCR

| **Primer Identity** | **Sequence (direction 5' to 3')** |
| --- | --- |
| bc_1002_For | /5phos/ACACACAGACTGTGAGgcagtcgaacatgtagctgactcaggtcac |
| bc_1005_For | /5phos/CACTCGACTCTCGCGTgcagtcgaacatgtagctgactcaggtcac |
| bc_1006_For | /5phos/CATATATATCAGCTGTgcagtcgaacatgtagctgactcaggtcac |
| bc_1007_For | /5phos/TCTGTATCTCTATGTGgcagtcgaacatgtagctgactcaggtcac |
| bc_1010_Rev | /5phos/CTCTGAGATAGCGCGTtggatcacttgtgcaagcatcacatcgtag |
| bc_1011_Rev | /5phos/ATAGATATACGTATAGtggatcacttgtgcaagcatcacatcgtag |
| bc_1012_Rev | /5phos/ACACGCGATCTAGTGTtggatcacttgtgcaagcatcacatcgtag |
| bc_1013_Rev | /5phos/CTCGCGTATGCGAGAGtggatcacttgtgcaagcatcacatcgtag |
| bc_1014_Rev | /5phos/ACGCGCGCGTAGTGAGtggatcacttgtgcaagcatcacatcgtag |
| bc_1015_Rev | /5phos/ACACACGTGTCATGCGtggatcacttgtgcaagcatcacatcgtag |
| bc_1016_Rev | /5phos/ATACTATCTCTCTATGtggatcacttgtgcaagcatcacatcgtag |
