# Supplementary material for: Allele mining, amplicon sequencing and computational prediction of Solanum melongena L. FT/TFL1 gene homologs uncovers putative variants associated to seed dormancy and germination
Source: PLoS One. 2023 May 3;18(5):e0285119. doi: 10.1371/journal.pone.0285119 (PMC10156061; doi:10.1371/journal.pone.0285119)
Supplement: S3 Table — (DOCX) [file pone.0285119.s005.docx]

**Table S3**. Primer combinations used in the second round of PCR based on cultivars

| **Cultivar** | **Plant Sample** | **Forward Primer** | **Reverse Primer** |
| --- | --- | --- | --- |
| Surya | Sample1 | bc_1006_For | bc_1010_Rev |
|  | Sample2 | bc_1006_For | bc_1011_Rev |
|  | Sample3 | bc_1006_For | bc_1012_Rev |
| EP-47 Annamalai | Sample1 | bc_1005_For | bc_1012_Rev |
|  | Sample2 | bc_1005_For | bc_1013_Rev |
|  | Sample3 | bc_1005_For | bc_1014_Rev |
| Pant Samrat | Sample1 | bc_1007_For | bc_1014_Rev |
|  | Sample2 | bc_1007_For | bc_1015_Rev |
|  | Sample3 | bc_1007_For | bc_1016_Rev |
| Arka Nidhi | Sample1 | bc_1002_For | bc_1013_Rev |
|  | Sample2 | bc_1002_For | bc_1014_Rev |
|  | Sample3 | bc_1002_For | bc_1015_Rev |
