# Supplementary material for: Allele mining, amplicon sequencing and computational prediction of Solanum melongena L. FT/TFL1 gene homologs uncovers putative variants associated to seed dormancy and germination
Source: PLoS One. 2023 May 3;18(5):e0285119. doi: 10.1371/journal.pone.0285119 (PMC10156061; doi:10.1371/journal.pone.0285119)
Supplement: S4 Table — (DOCX) [file pone.0285119.s006.docx]

**Table S4.** Distribution of unmutated *SmMFT*-2 alleles in the mutant populations of cultivars, Surya, EP-47 Annamalai, Pant Samrat and Arka Nidhi.

|  | | | **VI047336** | | | | | | |
| --- | --- | --- | --- | --- | --- | --- | --- | --- | --- |
| **Sample** | | **Subread Coverage** | | | **Amplicon Coverage** | | **Allele** | | |
| An_1-9 | | 476 | | | 184 | | *SmMFT*-2-*allele*1 | | |
| An_3-8 | | 473 | | | 173 | | *SmMFT*-2-*allele*1 | | |
| An_2-1 | | 475 | | | 404 | | *SmMFT*-2-*allele*1 | | |
| An_6-1 | | 463 | | | 222 | | *SmMFT*-2-*allele*1 | | |
| An_N3 | | 306 | | | 81 | | *SmMFT*-2-*allele*1 | | |
| An_1-7 | | 462 | | | 160 | | *SmMFT*-2-*allele*1 | | |
| An_6-8 | | 199 | | | 27 | | *SmMFT*-2-*allele*1 | | |
| An_N4-4 | | 473 | | | 124 | | *SmMFT*-2-*allele*1 | | |
| An5-11 | | 479 | | | 102 | | *SmMFT*-2-*allele*1 | | |
|  | | | **VI045550** | | | | | | |
| Sa_9-1  **Heterozygous**  Sa_9-1 | | 127  205 | | | 64  85 | | *SmMFT*-2*-allele*1  *SmMFT*-2-*allele*2 | | |
| Sa_B6-1  **Heterozygous**  Sa_B6-1 | | 294  191 | | | 88  60 | | *SmMFT2-allele*1  *SmMFT*-2-*allele*2 | | |
| Sa_3-1 | | 455 | | | 282 | | *MFT1-allele1* | | |
| Sa_4-2  **Heterozygous**  Sa_4-2 | | 134  345 | | | 34  65 | | *SmMFT*-2*-allele*1  *SmMFT*-2-*allele*2 | | |
| Sa_B7-2  **Heterozygous**  Sa_B7-2 | | 145  339 | | | 59  122 | | *SmMFT-2-allele*1  *SmMFT*-2-*allele*2 | | |
| Sa_B3-1 | | 480 | | | 112 | | *SmMFT*-2-*allele*1 | | |
| Sa_B1-2 | | 470 | | | 183 | | *SmMFT*-2-*allele*1 | | |
| Sa_7-2 | | 468 | | | 105 | | *SmMFT*-2-*allele*1 | | |
|  | | | **VI045276** | | | | | | |
| Ry_19-2 | | 471 | | | 182 | | *SmMFT*-2-*allele*1 | | |
| Ry_18-6 | | 479 | | | 184 | | *SmMFT*-2-*allele*1 | |  |
| Ry_31-3 | | 161 | | | 77 | | *SmMFT*-2-*allele*1 | |  |
| Ry-9-3 | | 485 | | | 160 | | *SmMFT*-2-*allele*1 | |  |
| Ry_36-5 | | 485 | | | 162 | | *SmMFT*-2-*allele*1 | |  |
| Ry-29-3 | | 479 | | | 142 | | *SmMFT*-2-*allele*1 | |  |
| Ry_26-4 | | 474 | | | 170 | | *SmMFT*-2-*allele*1 | |  |
| Ry_9-1 | | 101 | | | 66 | | *SmMFT*-2-*allele*1 | |  |
| Ry_5-1 | | 483 | | | 238 | | *SmMFT*-2-*allele*1 | |  |
| Ry_33-7 | | 481 | | | 177 | | *SmMFT*-2-*allele*1 | |  |
| Ry_31-6 | | 470 | | | 312 | | *SmMFT*-2-*allele*1 | | |
| Ry_33-5 | | 260 | | | 110 | | *SmMFT*-2-*allele*1 | | |
| Ry_2-1 | | 470 | | | 176 | | *SmMFT*-2-*allele*1 | | |
| Ry_16-4 | | 428 | | | 129 | | *SmMFT*-2-*allele*1 | | |
|  | | | **VI045274** | | | | | | |
| Ni_6-2 | | 476 | | | 164 | | *SmMFT*-2-*allele*1 | | |
| Ni_6-1 | | 461 | | | 180 | | *SmMFT*-2-*allele*1 | | |
| Ni_5-6 | | 43 | | | 9 | | *SmMFT*-2-*allele*1 | | |
| Ni_5-1 | | 234 | | | 16 | | *SmMFT*-2-*allele*1 | | |

Note: We have included sequences from mutant eggplant population here to make use of all available sequence data. However, the development of EMS-mutant plants is not covered in this article. Further information is available upon request.
